# Synthesis, Central Nervous System Activity and Structure-Activity Relationships of Novel 1-(1-Alkyl-4-aryl-4,5-dihydro-1*H*-imidazo)-3-substituted Urea Derivatives

**DOI:** 10.3390/molecules20033821

**Published:** 2015-02-26

**Authors:** Elżbieta Szacoń, Marzena Rządkowska, Agnieszka A. Kaczor, Ewa Kędzierska, Sylwia Fidecka, Dariusz Matosiuk

**Affiliations:** 1 Department of Synthesis and Chemical Technology of Pharmaceutical Substances with Computer Modeling Lab, Faculty of Pharmacy with Division of Medical Analytics, Medical University of Lublin, 4a Chodźki St., Lublin PL-20093, Poland; E-Mails: marzena.rzadkowska@umlub.pl (M.R.); agnieszka.kaczor@umlub.pl (A.A.K.); 2 School of Pharmacy, University of Eastern Finland, Yliopistonranta 1, P.O. Box 1627, Kuopio FI-70211, Finland; 3 Department of Pharmacology and Pharmacodynamics, Faculty of Pharmacy with Division of Medical Analytics, Medical University of Lublin, 4A Chodźki St., Lublin PL-20093, Poland; E-Mails: ewa.kedzierska@umlub.pl (E.K.); sylwia.fidecka@umlub.pl (S.F.)

**Keywords:** central nervous system activity, opioid receptors, urea derivatives

## Abstract

A series of 10 novel urea derivatives has been synthesized and evaluated for their central nervous system activity. Compounds **3a**–**3h** were prepared in the reaction between the respective 1-alkyl-4-aryl-4,5-dihydro-1*H*-imidazol-2-amines **1a** and **1b** and appropriate benzyl-, phenethyl-isocyanate or ethyl 4-isocyanatobenzoate and ethyl isocyanatoacetate **2** in dichloromethane. Derivatives **4c** and **4g** resulted from the conversion of **3c** and **3g** into the respective amides due to action of an aqueous ammonia solution. The results obtained in this study, based on literature data suggest a possible involvement of serotonin system and/or the opioid system in the effects of tested compounds, and especially in the effect of compound **3h**. The best activity of compound **3h** may be primarily attributed to its favourable ADMET properties, *i.e.*, higher lipophilicity (related to lower polar surface area and greater molecular surface, volume and mass than for other compounds) and good blood-brain permeation. This compound has also the greatest polarizability and ovality. The HOMO and LUMO energies do not seem to be directly related to activity.

## 1. Introduction

**Figure 1:**
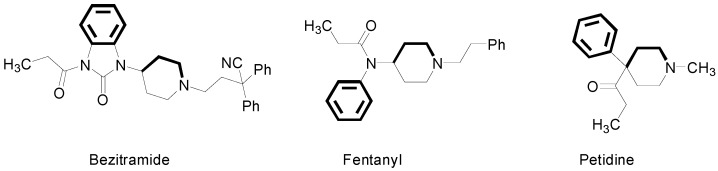
Structural formulas of bezitramide, fentanyl and petidine. Pharmacophoric features according to the Beckett’s model are shown in bold [2,5,6].

It is well known that many diseases are accompanied by inflammation and pain. Therefore, the search for new antinociceptive compounds is an important focus of attention for chemists as well as for pharmacologists [1,2]. Opioid receptors are key molecular targets for antinociceptive medications. Most morphine-like analgesics possess similar structural features, *i.e.*, the phenyl ring, tertiary nitrogen atom and the two carbon fragment (e.g., as a part of the piperidine ring), which are required by the receptor cavity [2,3,4]. These structural features are present in bezitramide, fentanyl and petidine, and their analogues (Figure 1) [2].

**Figure 2:**
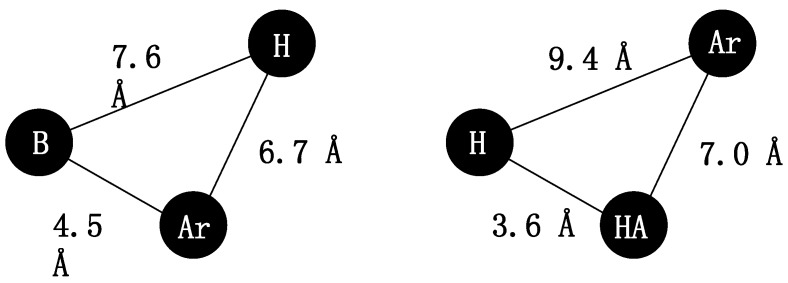
Non-classical opioid receptor pharmacophore models. B—base, Ar—aromatic; H—hydrophobic region, HA—hydrogen bond acceptor [2,5,6].

This “pharmacophore” model was elaborated by Beckett (with its subsequent modifications [7,8,9]) and was one of the first models used to explain the antinociceptive activity of morphine derivatives [2]. Later, non-classical pharmacophore models explaining opioid receptor activity were suggested, as presented in Figure 2. These models consist of a base (B), a hydrophobic (H) and aromatic moiety (Ar) or hydrogen bond acceptor (HA), hydrophobic (H), and aromatic groups (Ar) [7,10,11,12].

**Figure 3:**
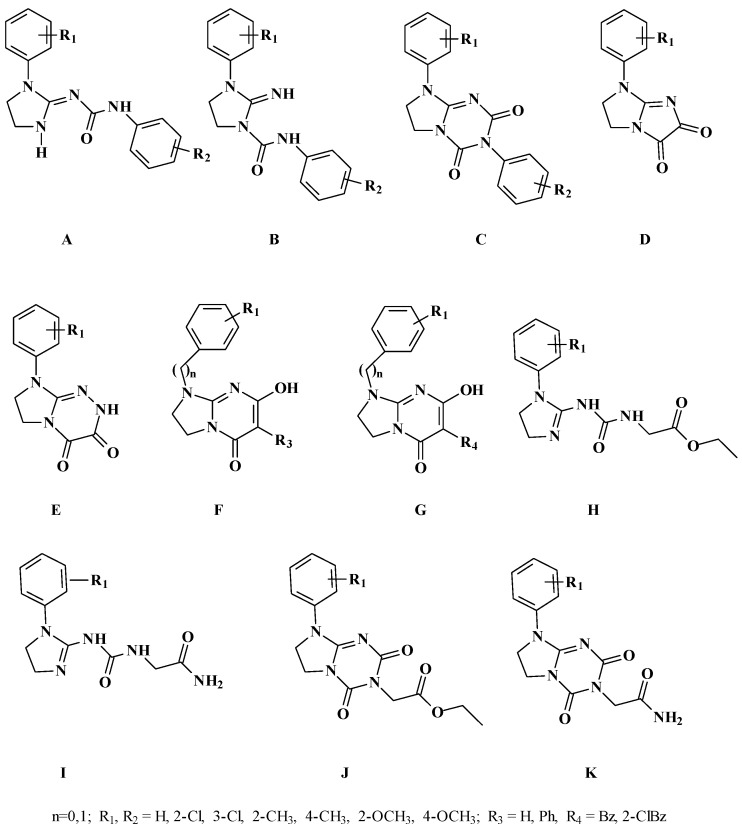
Previously reported antinociceptive and serotoninergic compounds. Series A–E and partially F exert their antinociceptive activity through the opioid system. Series G–K and some of compounds from series F have antinociceptive activity of unknown mechanism [2,5,6,10,11,12,13].

**Figure 4:**
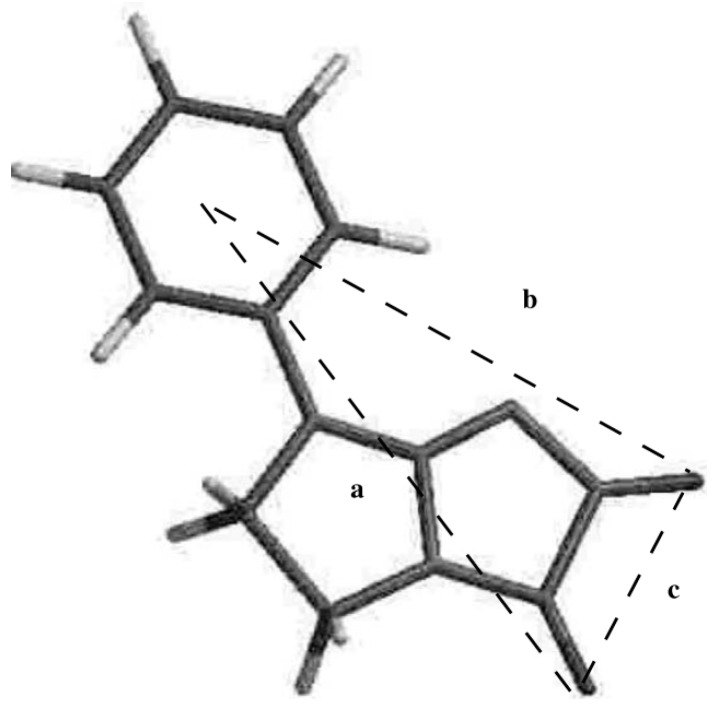
Pharmacophore model for the 5HT_2_ receptor [2,5,6,12].

Based on the non-classical pharmacophore models for opioid receptor activity we have previously reported a few series of compounds with antinociceptive activity mediated through the opioid system (series A–E [10,11,12,13]), partially mediated through opioid system (series F [2]) or with a different mechanism of antinociceptive activity (series G–K [5,6], Figure 3). Some of these compounds also exerted serotoninergic activity according to the pharmacophore model presented in Figure 4.

**Scheme 1:**
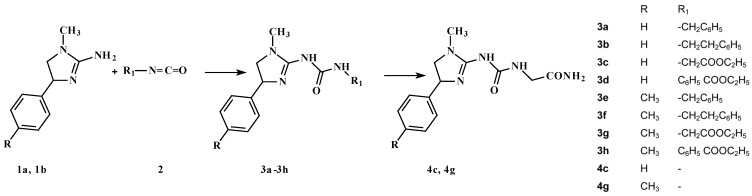
The synthesis scheme of the investigated compounds.

In our continuous effort towards the discovery of novel antinociceptive compounds with additional serotoninergic activity we have designed and synthesized a series of 10 N-substituted derivatives of 1-alkyl-4-ary(arylalkyl)imidazolidyn-2-ylideneureas **3a**–**3h**, **4c**, **4g** (Scheme 1). The rationale of this work can be summarized as follows: (1) the designed compounds follow the non-classical pharmacophore model for opioid receptor activity as well as the pharmacophore model for the serotoninergic activity; (2) the set of substituents in the aryl ring was selected on the basis of our earlier experience with the substituent effect on the activity. Here we present the synthesis, drug-likeness evaluation, ADMET prescreening, pharmacological studies for central nervous system activity and structure-activity relationship analysis for 10 N-substituted derivatives of 1-alkyl-4-ary(arylalkyl)-imidazolidyn-2-ylideneureas.

## 2. Results and Discussion

### 2.1. Chemistry

The synthetic route employed for the preparation of 1-(1-alkyl-4-aryl-4,5-dihydro-1*H*-imidazo)-3-substituted urea derivatives is shown in Scheme 1. We have previously reported a few series of 1-aryl-4,5-dihydro-1*H*-imidazol-2-amine derivatives which do not possess a protonable nitrogen atom but exhibit serotoninergic and antinociceptive activity mediated [2,10,11,12,13] or not [5,6] through the opioid system. Encouraged by these results, we designed, synthesized and studied compounds **3a**–**3h**, **4c**, **4g** (Scheme 1). Compounds **3a**–**3h** were prepared in the reaction between the respective 1-alkyl-4-aryl-4,5-dihydro-1*H*-imidazol-2-amines **1a** and **1b** and appropriate benzyl, phenethyl- isocyanate or ethyl 4-isocyanatobenzoate, ethyl isocyanatoacetate **2** in dichloromethane. Derivatives **4c** and **4g** resulted from the conversion of **3c** and **3g** into the respective amides due to the action of aqueous ammonia solution.

### 2.2. Estimation of Drug-Likeness

The descriptors applied for estimation of drug-likeness are presented in Table 1. Drug-likeness was assessed using Lipinski’s rule as well as the placement of the investigated compounds in the chemical space determined by the databases of the pharmacologically active compounds (CMC, Comprehensive Medicinal Chemistry Database, containing about 7000 compounds and MDDR, MACCS-II Drug Data Report, containing about 100,000 compounds) according to the methodology of PREADMET [14] service as described previously [5,6]. Concerning Lipinski’s rule, all the compounds possess the molar mass below 500, the number of hydrogen bond donors below five, the number of hydrogen bond acceptors below 10, and the lipophilicity below 5. Optimal value of lipophilicity for drugs acting on central nervous system is from 2 to 4 [15].

Regarding subsequent criteria of drug-likeness, most compounds collected in the CMC database has lipophilicity from −0.4 to 5.6, molar refractivity in the range of 40–130, molar mass from 160 to 480, and the number of atoms from 20 to 70 [5,6]. All the investigated compounds fulfill this criterion. Concerning the compounds in MDDR database, the drug-like substances have the number of rings equal or greater than 3, the number of rigid bonds equal or greater than 18, and the number of rotatable bonds equal or greater than 6 [5,6]. Compounds **3c**, **3g**, **4c** and **4g** possess too few rings. Compound **3c** has in addition too few rotatable bonds. Compounds **3a**, **3b**, **3e**, **3f**, **4c** and **4g** have too low a number of rotatable bonds which we will consider in the design of next series of compounds. Finally, molecule drug-likeness score (fragment-based score) was calculated using Osiris Property Explorer [16]. According to this score compounds **3a**, **3b**, **3c**, **3f**, **4c**, **4g** are more drug-like than the rest of compounds. In summary, the investigated compounds may be termed drug-like, and it is justified to test them in the *in vivo* experiments. Evaluation of drug-likeness has not allowed to exclude any compound from *in vivo* experiments. In particular compound **3h** has low drug-likeness score but it was selected for *in vivo* studies as the validation of *in silico* approach.

**Table 1:** Parameters for drug-likeness estimation. HBD—a number of hydrogen bond donors; HBA—a number of hydrogen bond acceptors.

Comp.	Molar Mass	AlogP	HBD	HBA	Number of Atoms	Molar Refractivity	Rings	Rigid Bonds	Rotatable Bonds	Druglikeness Score
**3a**	308.378	2.630	2	5	43	91.64	3	21	4	5.22
**3b**	322.404	2.951	2	5	46	96.24	3	19	5	5.74
**3c**	304.344	1.102	0	7	42	82.41	2	17	6	5.77
**3d**	366.414	2.827	2	7	49	102.91	3	23	6	1.30
**3e**	322.404	3.116	2	5	46	96.06	3	22	4	3.87
**3f**	336.431	3.437	2	5	49	100.67	3	22	5	4.39
**3g**	318.371	1.589	2	7	45	86.84	2	18	6	-1.23
**3h**	380.44	3.313	2	7	52	107.33	3	24	6	-0.14
**4c**	261.280	0.815	4	7	34	69.30	2	18	2	5.56
**4g**	275.306	1.301	4	7	37	73.72	2	19	2	4.16

### 2.3. Prediction of ADMET Properties

**Figure 5:**
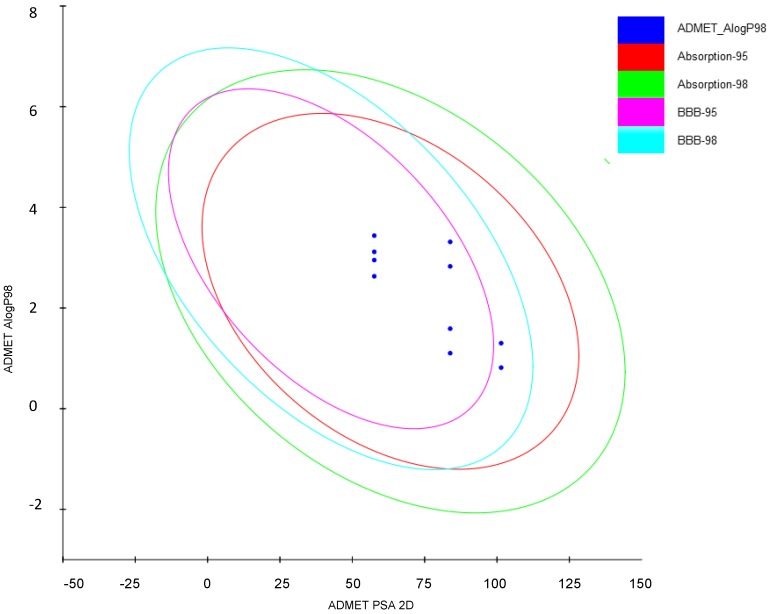
Evaluation of ADMET properties of the studied compounds.

In order to facilitate the selection of compounds for animal studies, some ADMET parameters were calculated (Table 2). The plot presented in Figure 5 confirms that most of the tested compounds possess favorable ADMET properties. Comparing the plot in Figure 5 with lipophilicity values from Table 1 and polar surface areas from Table 3, it can be concluded that compounds from series **4c** and **4g** have less favorable blood-brain permeation properties. All compounds are well absorbed (Figure 5), however compounds **3f** and **3h** are not enough soluble in water as they have values of logS below −4 [16]. Moreover, compounds **4c** and **4g** have lower overall drug score which combines drug-likeness, cLogP, LogS, molecular weight and toxicity risks in one convenient value than may be used to judge the compound’s overall potential to qualify as a drug [16]. Importantly, compounds from series **3a**–**3h** are predicted to be non-toxic (all scores equal to 1.00 in Table 2) whereas compounds from series **4c** and **4g** have middle risk (score 0.8) of mutagenic and tumorigenic properties and may have reproductive effects. On the basis of calculation of ADMET parameters and our earlier experience on the effect of substituents on the activity, we decided to test *in vivo* three compounds **3a**, **3h** and **4c**.

**Table 2:** ADMET parameters of the studied compounds. S—solubility.

Comp.	LogS	Toxicity Risk				Drug Score
		Mutagenic	Tumorigenic	Irritant	Reproductive Effective	
**3a**	−3.454	1.00	1.00	1.00	1.00	0.85
**3b**	−3.618	1.00	1.00	1.00	1.00	0.82
**3c**	−2.118	1.00	1.00	1.00	1.00	0.93
**3d**	−3.818	1.00	1.00	1.00	1.00	0.67
**3e**	−3.923	1.00	1.00	1.00	1.00	0.80
**3f**	−4.078	1.00	1.00	1.00	1.00	0.77
**3g**	−2.594	1.00	1.00	1.00	1.00	0.55
**3h**	−4.255	1.00	1.00	1.00	1.00	0.51
**4c**	−2.004	0.80	0.80	1.00	0.80	0.48
**4g**	−2.501	0.80	0.80	1.00	0.80	0.46

**Table 3:** Structural and electronic parameters of investigated compounds. PSA—polar surface area, HOMO—Highest Occupied Molecular Orbital, LUMO—Lowest Unoccupied Molecular Orbital.

Comp.	Surface Å^2^	PSA Å^2^	Volume Å^3^	Ovality	HOMO eV	LUMO eV	Polarizability	Molecular Weight
**3a**	586.1	61.2	288.6	1.652	−9.24	0.43	34.836	308.37
**3b**	573.1	65.0	304.7	1.706	−9.10	0.49	36.896	322.40
**3c**	589.8	107.7	278.8	1.702	−9.14	0.58	31.729	304.34
**3d**	660.9	104.3	328.4	1.734	−8.92	−0.65	39.549	366.41
**3e**	614.6	55.6	301.1	1.670	−9.20	0.42	36.854	322.40
**3f**	616.3	54.3	321.9	1.739	−9.07	0.53	38.789	336.43
**3g**	601.7	131.0	290.9	1.697	−9.26	0.38	33.862	318.37
**3h**	683.8	92.9	342.6	1.770	−9.00	−0.14	41.710	380.44
**4c**	482.5	170.8	225.8	1.556	−9.40	0.46	27.094	261.28
**4g**	523.3	175.1	245.2	1.630	−9.28	0.42	28.801	275.30

### 2.4. Pharmacological Activity

In this study we tested properties of three new 1-(1-alkyl-4-aryl-4,5-dihydro-1*H*-imidazo)-3-substituted urea derivatives: **3a**, **3h** and **4c**. Toxicity of tested compounds was 550 mg/kg ip for **3a**, 1300 mg/kg ip for **3h** and 800 mg/kg ip for **4c**, and therefore these ED_50_ values were adopted for further study. The spontaneous activity and amphetamine hyperactivity were evaluated. The effect on body temperature and behaviour of animals caused by administration of L-5-HTP, motor coordination as well as nociceptive and anticonvulsant activity were also estimated. These allowed preliminary determination of the impact of the new substances on the central nervous system (CNS) of experimental animals.

**Figure 6:**
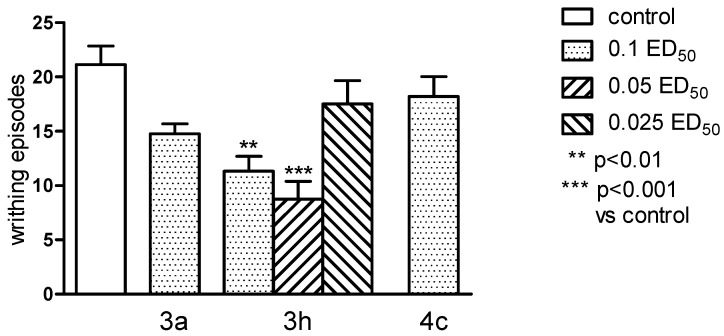
The antinociceptive effects of the tested compounds **3a**, **3h** and **4c**, assessed in the “writhing” test in mice. The results are expressed as mean ± SEM of a group of 8–10 mice. One-way ANOVA showed significant changes in the number of writhing episodes of mice after the administration of the compound **3h** (F_5,47_ = 5.734, *p* < 0.001). *Post hoc* Dunnett’s test confirmed a significant reduction in the writhing episodes of mice after the administration of the compound **3h** in doses of 0.1 and 0.05 ED_50_ (*p* < 0.01 and *p* < 0.001, respectively).

**Figure 7:**
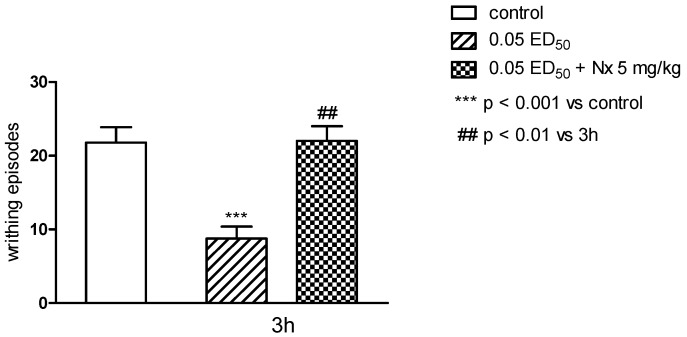
The influence of naloxone, 5 mg/kg s.c. on antinociceptive activity of compound **3h** evaluated in “writhing” test in mice. The results are expressed as mean ± SEM of a group of 6–14 mice. One-way ANOVA showed significant changes in the numer of writhing episodes of mice after the administration of the compound **3h** and coadministration of compound **3h** and naloxone (F_2,25_ = 11.42; *p* < 0.001). *Post hoc* Dunnett’s test confirmed a significant reduction in the writhing episodes of mice after the administration of the compound **3h** in the 0.05 ED_50_ dose (*p* < 0.001). Pretreatment with naloxone increased the numer of writhing episodes compared to compound 2 g group (*p* < 0.01).

The antinociceptive properties were tested by performing “writhing test”. This test is one of the most sensitive methods to determine the antinociceptive properties, and by its use it is possible to detect even very weak antinociceptive agents. It is also considered as an experimental model closest to the nature of clinical pain. It allows evaluation of analgesic action of both central and peripheral origin. However, this method introduces some limitations: in this test it is difficult to determine the length of antinociceptive activity, and the test is not specific as it can show an analgesic effect for many substances [17,18]. Only substance **3h** showed antinociceptive effect in this test and when administered both in higher dose (0.1 ED_50_) and at half dose (0.05 ED_50_) caused very clear and statistically significant (respectively *p* < 0.01 and *p* < 0.001) reduction in the number of writhing episodes in mice (Figure 6). In order to more closely determine the mechanism of antinociceptive activity, the writhing test was performed with the use of nonselective opioid antagonist—naloxone [19]. Naloxone very clearly and statistically significant (*p* < 0.01), decreased antinociceptive activity of the substance **3h** (Figure 7). This suggests the possibility of linking the activity of this compound with the opioid system, and also makes this compound a good predictor of the structure in the search for a new group of compounds acting through the opioid system.

**Figure 8:**
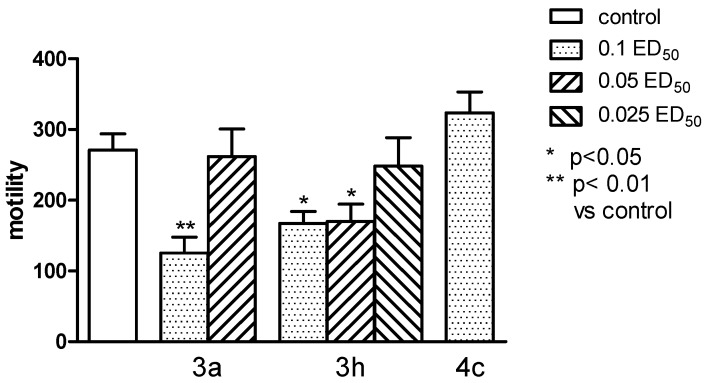
The influence of the tested compounds **3a**, **3h**, and **4c** on the spontaneous locomotor activity of mice. The results are expressed as mean ± SEM of a group of 6–14 mice. One-way ANOVA showed significant changes in locomotor activity of mice after the administration of compounds **3a** and **3h** (F_6,47_ = 6,204, *p* < 0.0001). *Post hoc* Dunnett’s test confirmed a significant reduction in motility of mice after the administration of the compound **3a** in the dose of 0.1 ED_50_ (*p* < 0.01) and **3h**—0.1 and 0.05 ED_50_ (*p* < 0.05).

**Figure 9:**
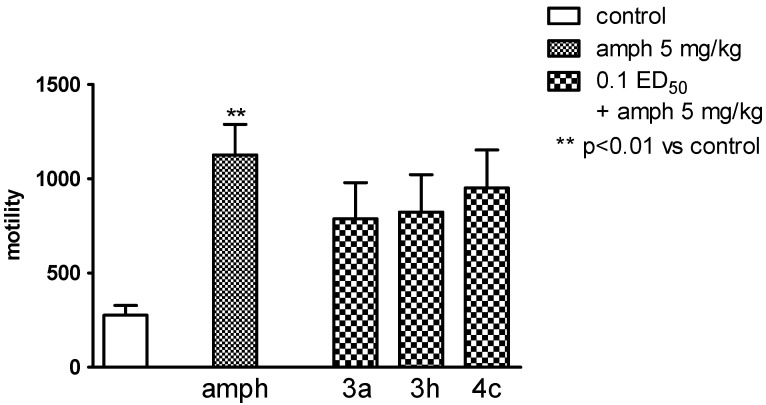
The influence of the tested compounds **3a**, **3h**, and **4c** on amphetamine-induced hyperactivity in mice. The results are expressed as mean ± SEM of a group of 7–8 mice. One-way ANOVA revealed significant changes in locomotor activity of mice after the administration of amphetamine (F_4,31_ = 3.885; *p* < 0.05). Simultaneous injection of each of the tested compounds with amphetamine did not change the activity of mice compared to amphetamine group.

Compound **3c** significantly reduced the locomotor activity of animals only in a dose of 0.1 ED_50_ (*p* < 0.01), and the compound **3h**—in the dose of 0.1 and 0.05 ED_50_ (*p* < 0.05) (Figure 8), while none of the tested compounds did not change the hyperactivity caused by administration of amphetamine (Figure 9).

Tests were also carried out to evaluate the effect of the new urea derivatives on the head-twitch responses (HTR) in mice caused by administration of a serotonin precursor, L-5-HTP, which may indicate the involvement of the serotonergic system in the observed effects. The head twitch response evoked in mice occurs as a result of increased activity of central 5-hydroxytryptamine (5-HT) neuronal systems [20]. This behavior appears to be mediated by 5-HT_2_ receptors. Several studies have established that direct and indirect 5-HT agonists induce HTR in rodents [20,21,22,23,24,25,26,27]. Furthermore, 5-HT_2_ receptor antagonists selectively block HTR [26,27,28,29,30], and their potency is highly correlated with the antagonist’s affinity for 5-HT_2_ receptors [27,31]. However, this test is not very specific because, other compounds such as adrenergic ligands, can change the HTR [32].

**Figure 10:**
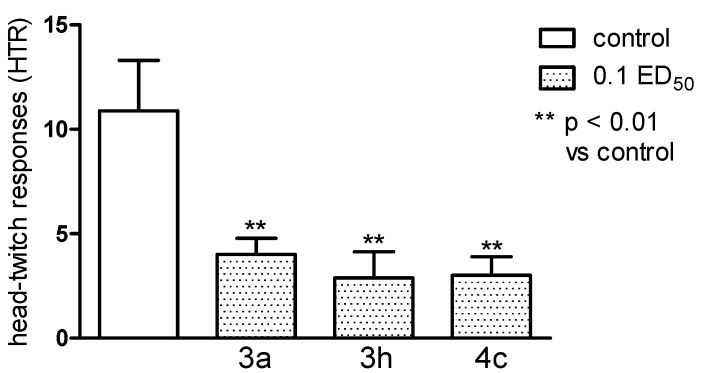
The influence of the tested compounds **3a**, **3h** and **4c** on the head-twitch responses (HTR) evoked by L-5-HTP (230 mg·kg^−1^). The results are expressed as mean ± SEM of a group of 8–10 mice. One-way ANOVA showed significant changes in the number of HTR (F_3,30_ = 6.902, *p* < 0.01). The *post hoc* Dunnett’s test confirmed a significant decrease in the numer of HTR after the administration of all of the tested compounds: **3a**, **3h** and **4c** in the dose of 0.1 ED_50_ (*p* < 0.01).

HTR to 5-HTP were significantly decreased by all the substances investigated (*p* < 0.01), from a mean of 12.2 ± 3.12 to 4.0 ± 0.86 by **3c**, 4.4 ± 2.2 by **3h** and 3.0 ± 0.89 by **4c** (Figure 10). The result seems to point out some connection with the 5-HT system.

**Figure 11:**
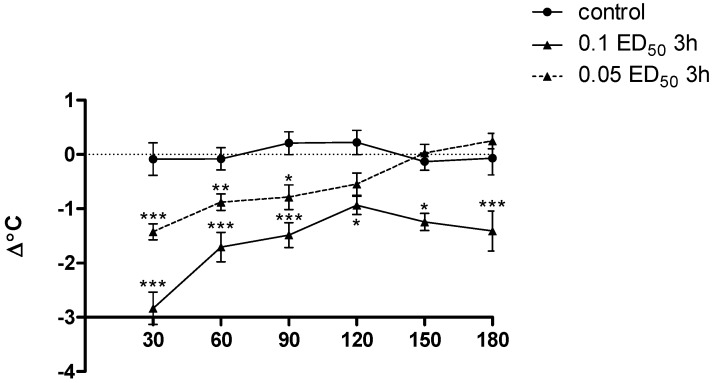
The influence of compound **3h** (used in dose of 0.1 and 0.05 ED_50_) on the body temperature of mice. Each point represents the mean for a group of 8–10 mice. Two-way ANOVA revealed significant effects for both dose [F_(2.153)_ = 73.38; *p* < 0.0001] and time [(F_(5.153)_ = 9.29; *p* < 0.0001], as well as a statically significant dose × time [F_(10.153)_ = 3.22; *p* < 0.001]. *Post hoc* Bonferroni test confirmed a significant decrease of the body temperature of mice after the administration of compound **3h** at the dose of 0.1 ED_50_ in 30, 60, 90, 180 min (*p* < 0.001), and from 120 to 150 min (*p* < 0.05) and at the dose of 0.05 ED_50_ in 30 min (*p* < 0.001), 60 min (*p* < 0.01) and 90 min (*p* < 0.05). * *p* < 0.05; ** *p* < 0.01; *** *p* < 0.001.

Only the substance **3h** had a significant effect on body temperature in mice: given in a dose corresponding to 0.1 ED_50_ caused a very clear, statistically significant and prolonged decrease in body temperature in mice (*p* < 0.001 from 30 to 90, and in 180 min, and *p* < 0.01 from 120 to 150 min). Moreover, administration of a lower dose of this substance (0.05 ED_50_) resulted in statistically significant reduction of the observed parameter, but this effect was somewhat lower and short-lived (*p* < 0.001 at 30 min only, and *p* < 0.01 from 60 to 90 min) (Figure 11). Administration of other compounds practically did not affect the body temperature of animals.

Serotonin has been reported to play an important role in central regulation of body temperature [33,34,35]. The MAO (monoamine oxidase) type A inhibitors appear to be crucially involved in hypothermia [36]. As a result of MAO-inhibition, 5-HT levels in the body are increased and may precipitate a serotonin syndrome. Hypothermia in rodents has been reported for MAO type A enzyme inhibitors (antidepressant drugs) such as clorgyline [37] and harman (1-methyl-β-carboline) [38]. In the pentetrazol seizure test, none of the tested compounds clearly reduced the severity of clonic or tonic seizures, or protected the animals from dying (data not presented).

**Figure 12:**
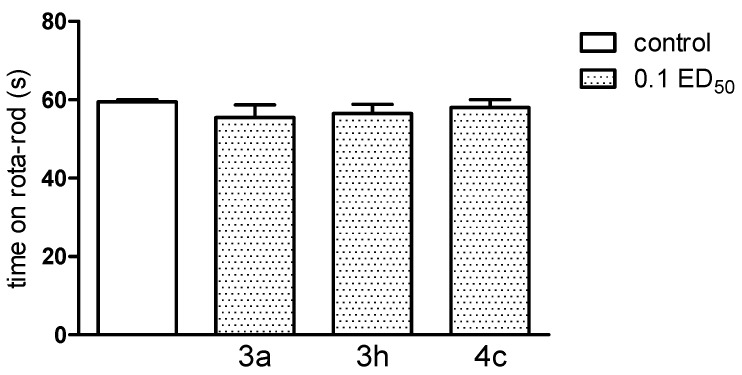
The influence of the tested compounds **3a**, **3h** and **4c** on motor coordination in the rota-rod test. The results are expressed as mean ± SEM of a group of 10 mice. One-way ANOVA did not show any significant changes in time spent on rota-rod [F_3,36_ = 0.61; *p* = 0.6129].

**Figure 13:**
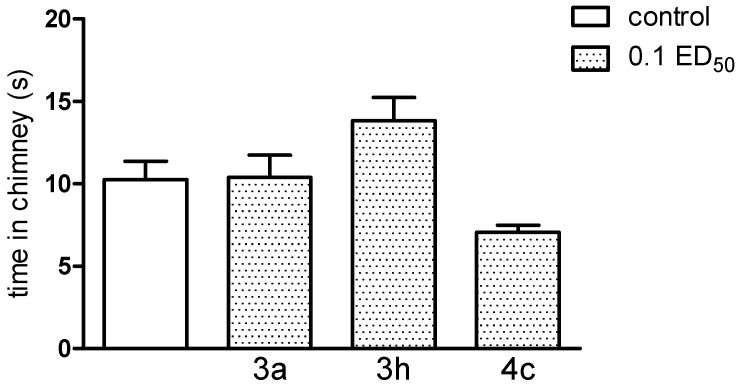
The influence of the tested compounds **3a**, **3h** and **4c** on motor coordination in the chimney test. The results are expressed as mean ± SEM of a group of 8–10 mice. None of the investigated compounds caused motor deficits in the chimney test. All animals were able to leave the chimney within less than 60 s.

It should be noted that new compounds, used at a dose of 0.1 ED_50_, caused no coordination disorders, as they did not change the behaviour of mice in either assay—the chimney, as well as the rota-rod test (Figure 12 and Figure 13). If present, impairment of motor coordination can express not only neurotoxicity, but can also influence on the results of the other tests, e.g., on the reaction to nociceptive stimuli of laboratory animals. Based on performed experiments, we cannot exclude any acute side effects of these compounds. Motility (after administration of compound **3a** and **3h** (only at the dose of 0.1 ED_50_) as well as body temperature of normothermic mice (after compound **3h**—at the dose of 0.1 and 0.05 ED_50_) were decreased, indicating some depressant action on the CNS.

The results of the pharmacological investigation showed that compounds tested exert significant influence on the CNS of laboratory animals. The observed effects seem to be connected primarily with serotonergic and/or opioid system. This involvement, however, is unclear and requires further study.

### 2.5. Structure-Activity Relationship

**Figure 14:**
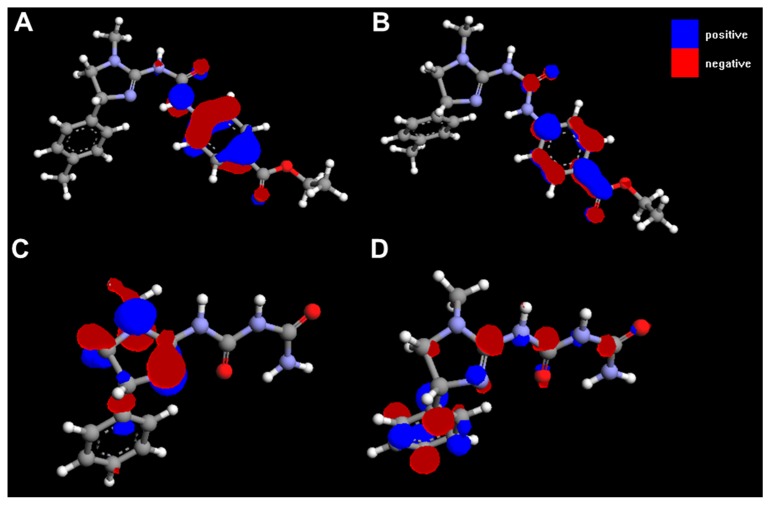
HOMO (**A**, **C**) and LUMO (**B**, **D**) for **3h** (A, B) and **4c** (C, D).

**Figure 15:**
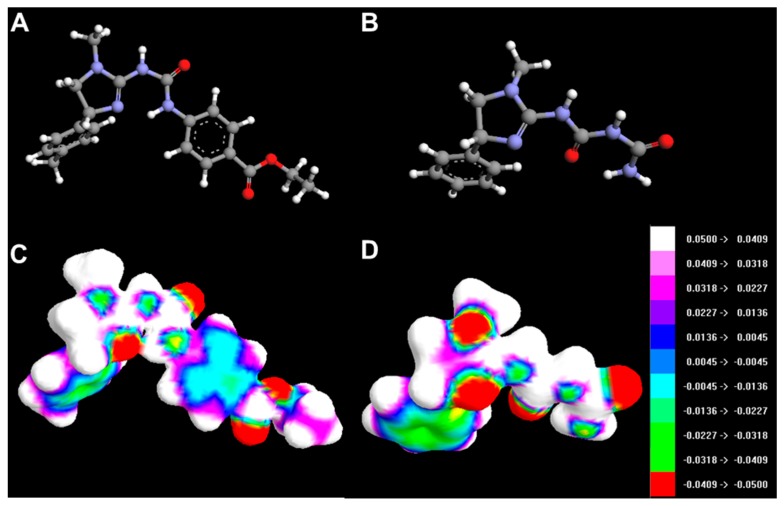
Molecular structures of 3h (**A**) and 4c (**B**). The map of the electrostatic potential (ESP) onto a surface of the electron density for 3h (**C**) and 4c (**D**).

HOMO and LUMO orbitals for selected compounds are shown in Figure 14. Molecular structures and electrostatic potential distribution of selected compounds are presented in Figure 15.

The best activity of compounds **3h** may be primarily attributed to its favourable ADMET properties, *i.e.*, higher lipophilicity (related to lower polar surface area and greater molecular surface, volume and mass than for other compounds, Table 3) and good blood-brain permeation. Ths compound has also the greatest polarizability and ovality. The HOMO and LUMO energies do not seem to be directly related to activity (Table 3).

## 3. Experimental Section

### 3.1. Chemistry

All commercial reagents and solvents were purchased from Sigma-Aldrich (Spruce, St. Louis, MO, USA) and used without purification. Reactions were routinely monitored by thin-layer chromatography (TLC) in silica gel (60 F_254_ plates Merck, Darmstadt, Germany) and the products were visualized with ultraviolet light of 254 nm wavelength. All NMR spectra were acquired on a Bruker AVANCE III 300 MHz spectrometer (Bruker, Billerica, MA, USA) equipped with BBO Z-gradient probe. Spectra were recorded at 25 °C using DMSO as a solvent with a non-spinning sample in 5 mm NMR-tubes. MS spectra were recorded on Bruker microTOF-Q II and processed using Compass Data Analysis software. The elementary analysis was performed with the application of Perkin-Elmer analyzer (940 Winter St., Waltham, MA, USA). Melting points were determined with a Boetius apparatus (Jena, Germany).

#### 3.1.1. General Procedure for the Synthesis of Compounds **3a**–**3h**

Benzyl/phenethylisocyanate or ethyl 4-isocyanatobenzoate, ethyl isocyanatoacetate **2** were dissolved in dichloromethane (25 mL) under atmosphere of dry nitrogen and added to a solution of the free base of 1-alkyl-4-aryl-4,5-dihydro-1*H*-imidazol-2-amines **1a**, **1e** (0.01 mol) dissolved in dichloromethane (100 mL). The mixture was shaken for 24 h at room temperature. Solvent was removed by distillation and the rubber-like residue was treated with warm propan-2-ol. The solid product was filtrated off and recrystallized from propan-2-ol.

*1-(1-Methyl-4-phenyl-4,5-dihydro-1H-imidazo)-3-benzylurea* (**3a**). From **1a** (1.75 g) **3a** (1.51 g, 49% yield) was obtained as a white crystalline solid, mp 145–146 °C; ^1^H-NMR (DMSO-*d*_6_): δ = 8.63 (s, 1H, NH); 8.01 (s, 1H, NH); 7.08–7.42 (m, 10H, H-Ar); 4.01–4.17 (d, 2H, C4, *J* = 7.6 Hz); 3.61 (s, 1H, C5), 3.48 (s, 2H, CH_2benzyl_); 2.24 (s, 3H, CH_3_); ^13^C-NMR (DMSO-d_6_): δ = 17.1 (CH_3_); 38.2 (CH_2_); 48.5 C5 (CH_2_); 68.9 C4 (CH); 161.0 (C=N); 171.0 (C=O); 119.1, 120.3, 120.7, 122.9, 124.2, 127.6 128.4, 129.1, 129.8 (C-Ar); EIMS *m*/*z* 309.1 [M+H]^+^. HREIMS (*m*/*z*): 308.1340 [M^+^]; (Calcd for C_18_H_20_N_4_O 308.3900); Anal. Calcd for: C_18_H_20_N_4_O; Anal. Found C, 70.35; H, 6.59; N, 17.17; Calcd C, 70.10; H, 6.35; N, 18.16.

*1-(1-Methyl-4-phenyl-4,5-dihydro-1H-imidazo)-3-phenethylurea* (**3b**). From **1a** (1.75 g) **3b** (1.06 g, 33% yield), was obtained as a white crystalline solid, mp 120–122 °C; ^1^H-NMR (DMSO-*d*_6_): δ = 8.56 (s, 1H, NH); 7.29 (s, 1H, NH); 6.89–7.63 (m, 10H, H-Ar); 4.00–4.10 (d, 2H, C4, *J* = 7.5 Hz); 3.45 (s, 1H, C5); 3.78–3.85 (m, 2H, CH_2phenethyl_); 3.17–3.24 (m, 2H, CH_2phenethyl_); 2.06 (s, 3H, CH_3_); ^13^C-NMR (DMSO-*d*_6_): δ = 18.4 (CH_3_); 41.9 (CH_2_); 45.2 (CH_2_); 49.96 C5 (CH_2_); 67.8 C4 (CH); 161.3 (C=N); 171.6 (C=O); 114.2, 120.4, 120.8, 126.5, 127.1, 128.2, 129.1, 131.4,131.8, (C-Ar); EIMS *m*/*z* 323.5 [M+H]^+^. HREIMS (*m*/*z*): 322.1921 [M^+^] (Calcd for C_19_H_22_N_4_O 322.4170); Anal. Calcd for: C_19_H_22_N_4_O: Anal. Found Found C, 70.65; H, 6.79; N, 17.49; Calcd C, 70.78; H, 6.87; N, 17.37.

*1-(1-Methyl-4-phenyl-4,5-dihydro-1H-imidazo)-3-(ethoxycarbonylmethyl)urea* (**3c**). From **1a** (1.75 g) **3c** (1.49 g, 48% yield) was obtained as a white crystalline solid, mp 159–161 °C; ^1^H-NMR (DMSO-*d*_6_): δ = 8.60 (s, 1H, NH); 8.27 (s, 1H, NH); 7.11–7.61 (m, 5H, H-Ar); 3.76 (s, 1H, C5), 4.02–4.15 (d, 2H, C4, *J* = 7.4 Hz); 3.98–4.02 (m, 2H, CH_2_); 3.71–3.82 (m, 2H, CH_2_); 2.30 (s, 3H, CH_3_); 1.26–1.31 (t, 3H, CH_3_, *J* = 7.1 Hz) ^13^C-NMR (DMSO-*d*_6_): δ = 17.9 (CH_3_); 19.3 (CH_3_); 41.3 (CH_2_); 43.6 (CH_2_); 49.6 C5 (CH_2_); 68.1 C4 (CH); 160.9 (C=N); 171.3 (C=O); 119.1, 120.4, 120.9, 123.5, 127.4, 129.1, 129.8, 132.5, 132.8, 133.5 (C-Ar); EIMS *m*/*z* 305.3 [M+H]^+^. HREIMS (*m*/*z*): 304.16730 [M^+^] (Calcd for C_15_H_20_N_4_O_3_ 304.3570); Anal. Calcd for: C_15_H_20_N_4_O_3_; Anal. Found C, 59.35; H, 6.29; N, 18.38; Calcd C, 59.20; H, 6.22; N, 18.41.

*1-(1-Methyl-4-phenyl-4,5-dihydro-1H-imidazo)-3-(4-ethoxycarbonylphenyl)urea* (**3d**). From **1a** (1.75 g) **3d** (2.82 g, 77% yield) was obtained as a white crystalline solid, mp 170–172 °C; ^1^H-NMR (DMSO-*d*_6_): δ = 9.05 (s, 1H, NH); 8.16 (s, 1H, NH); 7.05–7.68 (m, 9H, H-Ar); 4.01–4.18 (d, 2H, C4, *J* = 7.6 Hz); 3.29 (s, 1H, C5); 2.73–2.90 (m, 2H, CH_2_); 1.33 (s, 3H, CH_3_); 1.15–1.28 (t, 3H, CH_3,_ *J* = 7.0 Hz); ^13^C-NMR (DMSO-*d*_6_): δ = 18.3 (CH_3_); 21.3 (CH_3_); 41.3 CH_2_; 41.5 C5 (CH_2_); 69.3 C4 (CH); 161.2 (C=N); 171.7 (C=O); 170.9 (C=O); 117.6, 119.7, 128.3, 129.7, 131.3, 131.6, 133.5, 134.9, 136.1, 136.7 (C-Ar); EIMS *m*/*z* 367.1 [M+H]^+^. HREIMS (*m*/*z*): 366.1120 [M^+^] (Calcd for C_20_H_22_N_4_O_3_ 366.4280); Anal. Calcd for: C_20_H_22_N_4_O_3_; Anal. Found C, 65.45; H, 6.19; N, 15.38; Calcd C, 65.56; H, 6.05; N, 15.29.

*1-[1-Methyl-4-(methylphenyl)-4,5-dihydro-1H-imidazo]-3-benzylurea* (**3e**). From **1b** (1.89 g) **3e** (1.96 g, 61% yield) was obtained as a white crystalline solid, mp 132–134 °C; ^1^H-NMR (DMSO-*d*_6_): δ = 8.73 (s, 1H, NH); 8.04 (s, 1H, NH); 7.03–7.51 (m, 9H, H-Ar); 3.63 (s, 1H, C5), 4.02–4.18 (d, 2H, C4, *J* = 7.6 Hz); 3.85 (s, 2H, CH_2benzyl_); 2.29 (s, 3H, CH_3_); 1.63 (s, 3H, CH_3_); ^13^C-NMR (DMSO-*d*_6_): δ = 18.8 (CH_3_); 20.8 (CH_3_); 41.3 (CH_2_); 43.6 (CH_2_); 49.6 C4 (CH_2_); 68.4 C5 (CH); 162.5 (C=N); 172.0 (C=O); 119.2, 121.4, 121.9, 123.5, 128.2, 129.5, 130.8, 133.5, 133.8, 135.3 (C-Ar); EIMS *m*/*z* 319.1 [M+H]^+^. HREIMS (*m*/*z*): 318.2630 [M^+^] (Calcd for C_19_H_22_N_4_O 322.7050); Anal. Calcd for: C_19_H_22_N_4_O; Anal. Found C, 70.65; H, 6.69; N, 17.42; Calcd C, 70.71; H, 6.87; N, 17.38.

*1-[1-Methyl-4-(4-methylphenyl)-4,5-dihydro-1H-imidazo)-3-phenethylurea* (**3f**). From **1b** (1.89 g) **3f** (2.05 g, 61% yield) was obtained as a white crystalline solid, mp 116–117 °C; ^1^H-NMR (DMSO-*d*_6_): δ = 8.81 (s, 1H, NH); 8.54 (s, 1H, NH); 7.19–7.61 (m, 9H, H-Ar); 3.81 (s, 1H, C5), 4.09–4.19 (d, 2H, C4, *J* = 7.5 Hz); 3.61–3.76 (m, 2H, CH_2phenethyl_); 3.11–3.37 (m, 2H, CH_2phenethyl_); 2.30 (s, 3H, CH_3_); ^13^C-NMR (DMSO-*d_6_*): δ = 17.6 (CH_3_); 21.8 (CH_3_); 41.2 (CH_2_); 43.6 (CH_2_); 49.6 C4 (CH_2_); 69.2 C5 (CH); 160.8 (C=N); 172.8 (C=O); 120.0, 127.3, 120.5, 122.7, 127.2, 129.1, 129.4, 131.0, 131.9, 133.5 (C-Ar); EIMS *m*/*z* 337.3 [M+H]^+^. HREIMS (*m*/*z*): 336.3210 [M^+^] (Calcd for C_20_H_24_N_4_O 336.4440); Anal. Calcd for: C_20_H_24_N_4_O; Anal. Found C, 71.55; H, 7.32; N, 16.68; Calcd C, 71.40; H, 7.19; N, 16.65.

*1-[1-Methyl-4-(4-methylphenyl)-4,5-dihydro-1H-imidazo]-3-(ethoxycarbonylmethyl)urea* (**3g**). From **1b** (1.89 g) **3g** (1.56 g, 49% yield) was obtained as a white crystalline solid, mp 183–185 °C; ^1^H-NMR (DMSO-*d*_6_): δ = 9.04 (s, 1H, NH); 8.33 (s, 1H, NH); 7.02–7.64 (m, 4H, H-Ar); 3.49 (s, 1H, C5), 4.04–4.20 (d, 2H, C4, *J* = 7.5 Hz); 3.98–4.02 (m 2H, CH_2_); 3.51–3.62 (m 2H, CH_2_); 2.45 (s, 3H, CH_3_); 2.01 (s, 3H, CH_3_); 1.28–1.39 (t, 3H, CH_3_, *J* = 7.2) ^13^C-NMR (DMSO-*d*_6_): δ = 17.9 (CH_3_); 20.5 (CH_3_); 22.9 (CH_3_); 41.0 (CH_2_); 43.6 (CH_2_); 48.6 C5 (CH_2_); 67.1 C4 (CH); 161.5 (C=N); 171.9 (C=O); 112.3, 112.3, 112.8, 119.5, 120.9, 121.1, 128.7, 130.5, 131.8, 132.0 (C-Ar); EIMS *m*/*z* 319.1 [M+H]^+^. HREIMS (*m*/*z*): 318.1560 [M^+^] (Calcd for C_16_H_22_N_4_O_3_ 318.3840); Anal. Calcd for: C_16_H_22_N_4_O_3_; Anal. Found C, 60.45; H, 6.59; N, 17.38; Calcd C, 60.36; H, 6.97; N, 17.60.

*1-[1-Methyl-4-(4-methylphenyl)-4,5-dihydro-1H-imidazo]-3-(4-ethoxycarbonylphenyl)urea* (**3h**). From **1b** (1.89 g) **3h** (2.51 g, 66% yield) was obtained as a white crystalline solid, mp 154–156 °C; ^1^H-NMR (DMSO-*d*_6_): δ = 9.18 (s, 1H, NH); 8.44 (s, 1H, NH); 7.18–7.60 (m, 8H, H-Ar); 4.09–4.20 (d, 2H, C4, *J* = 7.4 Hz); 3.56 (s, 1H, C5); 2.79–2.81 (m, 2H, CH_2_); 1.97 (s, 3H, CH_3_); 1.33 (s, 3H, CH_3_); 1.12–1.25 (t, 3H, CH_3_); ^13^C-NMR (DMSO-*d*_6_): δ = 18.2 (CH_3_); 21.1 (CH_3_); 22.0 (CH_3_); 40.2 (CH_2_); 49.6 C5 (CH_2_); 68.0 C4 (CH); 162.1 (C=N); 171.5 (C=O); 119.9, 120.2, 121.6, 122.5, 123.4, 128.1, 122.8, 127.1, 127.8, 129.0 (C-Ar); EIMS *m*/*z* 381.4 [M+H]^+^. HREIMS (*m*/*z*): 380.3510 [M^+^] (Calcd for C_21_H_24_N_4_O_3_ 380.4550); Anal. Calcd for: C_21_H_24_N_4_O_3_; Anal. Found C, 66.45; H, 6.59; N, 14.81; Calcd C, 66.30; H, 6.36; N, 14.73.

#### 3.1.2. General Procedure to Obtain Compounds **4c**, **4g**

1-[1-Methyl-4-(alkyl)phenyl-4,5-dihydro-1*H*-imidazo]-3-(ethoxycarbonylmethyl)ureas **3c**, **3g** (0.01 mol) were dissolved in methanol (50 mL) and added to 20% aqueous ammonia solution. The mixture was shaken for 6 h at room temperature. The solid product was filtrated off and recrystallized from propan-2-ol.

*1-(1-Methyl-4-phenyl-4,5-dihydro-1H-imidazo)-3-aminocarbonylmethylurea* (**4c**). From **3c** (3.04 g) **4c** (1.65 g, 60% yield) was obtained as a white crystalline solid, mp 142–144 °C; ^1^H-NMR (DMSO-*d*_6_): δ = 9.62 (s, 1H, NH); 8.46 (s, 1H, NH); 8.11–8.16 (d, 2H, NH_2_); 7.40–7.69 (m, 5H, H-Ar); 3.89 (s, 1H, C4), 4.03–4.17 (d, 2H, C5, *J* = 7.5 Hz); 3.98–4.02 (m 2H, CH_2_); 2.30 (s, 3H, CH_3_); ^13^C-NMR (DMSO-*d*_6_): δ = 23.8 (CH_3_); 41.3 (CH_2_); 49.9 C5 (CH_2_); 69.8 C4 (CH); 160.3 (C=N); 171.3 (C=O); 172.5 (C=O); 119.3, 120.3, 121.6, 125.5, 125.9, 128.1, 129.7, 129.9 (C-Ar); EIMS *m*/*z* 276.3 [M+H]^+^. HREIMS (*m*/*z*): 275.3210 [M^+^] (Calcd for C_13_H_17_N_5_O_4_ 275.3190); Anal. Calcd for: C_13_H_17_N_5_O_4_; Anal. Found C, 56.45; H, 6.39; N, 25.38; Calcd C, 56.71; H, 6.22; N, 25.43.

*1-[1-Methyl-4-(4-methylphenyl)-4,5-dihydro-1H-imidazo]-3-aminocarbonylmethylurea* (**4g**). From **3g** (3.18 g) **4g** (1.33 g, 46% yield) was obtained as a white crystalline solid, mp 128–130 °C; ^1^H-NMR (DMSO-*d*_6_): δ = 9.23 (s, 1H, NH); 8.61 (s, 1H, NH); 8.05–8.11 (d, 2H, NH_2_); 6.76–7.38 (m, 4H, H-Ar); 3.73 (s, 1H, C5), 4.03–4.18 (d, 2H, C4, *J* = 7.6 Hz); 3.98–4.02 (m 2H, CH_2_); 2.30 (s, 3H, CH_3_); 1.93 (s, 3H, CH_3_); ^13^C-NMR (DMSO-*d*_6_): δ = 17.5 (CH_3_); 23.4 (CH_3_); 40.2 (CH_2_); 45.0 C5 (CH_2_); 68.5 C4 (CH); 160.7 (C=N); 172.1 (C=O); 169.8 (C=O); 118.3, 118.4, 120.1, 127.0, 127.9, 128.1, 129.7, 131.1 (C-Ar); EIMS *m*/*z* 290.5 [M+H]^+^. HREIMS (*m*/*z*): 289.1330 [M^+^] (Calcd for C_14_H_19_N_5_O_2_ 289.3460); Anal. Calcd for: C_14_H_19_N_5_O_2_; Anal. Found C, 58.23; H, 6.52; N, 24.38; Calcd C, 58.11; H, 6.61; N, 24.21.

### 3.2. Pharmacology

The experiments were performed on male Albino Swiss mice (18–30 g). The animals were kept 8–10 to a cage, at room temp. of 20 ± 1 °C, on a 12:12 h dark-light cycle. Standard food (LSM, Motycz, Poland) and water were available *ad libitum*. All experiments were performed between 9:00 a.m. and 4:00 p.m. The experiments were performed in accordance with the opinion of Local Ethics Committee for Animal Experimentation.

The investigated substances, **3c**, **3h** and **4c**, were administered intraperitoneally (i.p.) or subcutaneously (s.c.) in volume of 10 mL·kg^−1^ as suspensions in aqueous solution of 0.5% methylcellulose (tylose). The compounds were injected 60 min before the tests. The controls received the equivalent volume of the solvent. All tests performed, suggested by Vogel and Vogel [18], are generally accepted as basic in investigation of the central activity by behavioral methods. The acute toxicity of the compound was assessed in mice according to Litchfield and Wilcoxon method [39], as the ED_50_ calculated as “the lost of righting reflex” within 48 h. The compounds were injected in doses equivalent to 0.1, 0.05 and 0.025 ED_50_.

In addition, the activity of compounds was assessed in the following test: (1) locomotor activity was measured in photoresistor actometers for single mice for 30 min as spontaneous activity and amphetamine-induced hyperactivity: mice received subcutaneusly (s.c.) 5 mg/kg of amphetamine 30 min before the test; (2) nociceptive reactions were studied in the acetic acid (0.6%) induced writhing test [40]. The number of writhing episodes was measured for 10 min starting 5 min after i.p. administration of acid solution; (3) motor coordination was evaluated in rota rod test: [41] motor impairments, defined as the inability to remain on the rotating rod for 1 min were measured and the mean time spent on the rota-rod was counted for each mouse) and chimney test: [42] motor impairments were indicated by the inability to perform the test within 1 min); (4) body temperature in normothermic mice was measured in the rectum by thermistor thermometer; (5) pentylenetetrazole (110 mg/kg, s.c.)-induced convulsions were evaluated as the number of mice with clonic seizures, tonic convulsions and dead animals; (6) head twitch responses (HTR) after 5-hydroxytryptophan (L-5-HTP), were estimated acc. to Corne *et al.* [43]. Mice received L-5-HTP (230 mg/kg, i.p.) and the number of HTR was recorded in 6 two-minutes intervals (4–6, 14–16, 24–26, 34–36, 44–46, 54–56 min).

The obtained data were calculated by Fisher exact test (pentylenetetrazole-induced seizures), two-way analysis of variance (ANOVA) and followed by a *post hoc* confirmation with the Bonferroni test (body temperature), and one-way ANOVA followed by Dunnett’s *post hoc* test (other tests).

### 3.3. Molecular Modeling

The compounds investigated were modeled using the LigPrep protocol from the Schrödinger Suite [44]. In order to sample different protonation states of ligands in physiological pH, Epik module was used [45]. Parameters to evaluate drug-likeness were calculated using VegaZZ v. 3.0.1 [46] (number of atoms), Discovery Studio v. 3.1. [47] (molar mass, number of rings, lipophilicity, number of rotatable bonds), ACDLabs (molar refractivity, number of hydrogen bond donors and acceptors), and the Schrödinger Suite (a number of rigid bonds) as described previously [2,12,13]. Drug-likeness was also evaluated with Osiris Property Explorer [16]. This approach is based on a list of about 5300 distinct substructure fragments with associated drug-likeness scores. The drug-likeness is calculated summing up score values of those fragments that are present in the molecule under investigation. ADMET parameters were calculated with Discovery Studio 3.1 (solubility, blood-brain permeation) or Osiris Property Explorer [16] (toxicity risks). The prediction of toxicity by this tool relies on a precomputed set of structural fragment that give rise to toxicity alerts in case they are encountered in the investigated structure. For structure–activity relationship studies, HOMO and LUMO energies and polarizabilty were calculated with Discovery Studio 3.1 and molar surface, volume, ovality and polar surface area were calculated with VegaZZ as reported earlier [2,12,13]. The maps of the electrostatic potential (ESP) onto a surface of the electron density were visualized with ArgusLab [48].

## 4. Conclusions

In conclusion, we have designed and studied 10 compounds and one of them exhibited antinociceptive properties probably connected with the opioid system accompanied by serotoninergic properties. Further studies are necessary to investigate the molecular mechanism for this compound which will enable to apply structure-based design methods to obtain more favorable modifications.
